# Early Holocene Quantitative Summer Temperature Reconstructions in SE Lithuania Inferred from Chironomidae Data

**DOI:** 10.3390/biology14121692

**Published:** 2025-11-27

**Authors:** Neringa Gastevičienė, Gražyna Kluczynska, Vaida Šeirienė

**Affiliations:** State Scientific Research Institute Nature Research Centre, Akademijos 2, LT-08412 Vilnius, Lithuania; neringa.gasteviciene@gamtc.lt (N.G.); grazyna.kluczynska@gamtc.lt (G.K.)

**Keywords:** climate dynamics, July temperature, palaeoenvironment, lake sediments, Eastern Baltic

## Abstract

Chironomid subfossils were analysed at the Čepkeliai palaeolake in southern Lithuania in order to reconstruct the environmental history of the site during the Early Holocene, paying particular attention to climate change. Mean July air temperatures were inferred using two different chironomid–climate calibration data sets. As currently no chironomid-based climate calibration set is available from Lithuania, calibration data sets from Fennoscandia, Finland, and Poland were used instead. Our reconstructions suggest that mean July temperatures varied between 13.2 °C and 18.5 °C during the Early Holocene, similar to present-day temperatures. Both reconstructions show a series of climatic events that occurred during the Early Holocene, including the Preboreal oscillation and the “10.2”, “9.2”, and “8.2” oscillations. The reconstruction results correlate well with those carried out simultaneously in neighbouring countries, providing valuable information for this period, as such studies are still rare in the Eastern Baltic region.

## Simple Summary

In order to determine the causes of recent global warming and predict future trends, it is essential to examine how this process has occurred in the past. We used Chironomidae assemblage studies to reconstruct the environment and climate changes during the Early Holocene (11,700–8200 cal yrs BP). Subfossil chironomids were studied in palaeolake sediments in southern Lithuania. Our reconstructions suggest that the mean temperature in July varied between 13.2 and 18.5 °C, enabling us to identify three short and rapid climate oscillations during the period under study. Cool climate oscillations recorded in our study have also been registered in other regional records. Comparing these events makes it possible to identify general climate change trends and local environmental change patterns. Although temperature is a decisive factor in chironomid development, this study shows that local environmental conditions, such as water level, eutrophication, and pH changes, can sometimes determine the composition of chironomid assemblages.

## 1. Introduction

The recent acceleration of climate warming has prompted widespread concern and initiated the search for reliable methods to predict future scenarios of climate dynamics. Within this context, the study of past climate changes is of great importance. It provides the opportunity to assess modern climate dynamics in a temporal and spatial perspective and to improve our understanding of the causes of climate change. Lake sediments containing the remains of various organisms preserved in them are the major source of such proxy-climate data.

A wide range of proxies and techniques are used for past climate studies. The most common reconstructions are based on pollen data. However, it is important to acknowledge the potential occurrence of delays in vegetation’s response to rapid and short-term climate alterations [1,2,3]. This is especially important for the Holocene period, when climate fluctuations were less pronounced and changes in temperature were relatively small (<2°) and short-term (<100 years) [4]. Such temperature changes may not have been large enough to cause major vegetation shifts [5]. During the last decades among the numerous other proxy-indicators, non-biting midges (Insecta: Diptera: Chironomidae) emerged as one of the most reliable proxies for past climate reconstructions [2,6,7,8,9]. The head capsules of larvae are present in all types of water basins and are well preserved in sediments. The short life cycle, wide range of ecological niches, rapid response to climate change, sensitivity to prevailing temperature conditions, and methodological advancement, particularly improvements in fossil taxonomy, have rendered fossil chironomids one of the most widely used biological proxies in palaeoclimatology [1,10]. Many of the taxa are stenothermic, and because of these attributes the group has considerable potential as a proxy indicator of palaeoclimatic change (e.g., [11]). Also, since chironomids are sensitive to other environmental conditions such as pH, water level fluctuations, etc., palaeoecological analysis of sub-fossil chironomid assemblages can be used to reconstruct past palaeoenvironmental changes [12,13]. In this connection it has long been debated whether other environmental factors would affect temperature reconstructions (e.g., [7,14,15,16,17]). However, chironomid training data sets have confirmed that the primary control of the distribution of Chironomidae larvae is summer air and lake-water temperatures [11,18,19,20,21]. Air temperature is an important factor influencing the spread and reproduction of adults [9,22]. Meanwhile, the reproductive success of chironomid larvae is directly affected by water temperature [23]. Thus, they provide an important function as an indicator of past climate independent of other palaeoclimate proxies [24,25].

Quantitative temperature reconstructions from fossil chironomid assemblages are made by applying a transfer function that relates modern chironomid assemblages from surface sediments from a training set of lakes with the temperature at those sites. Although inference models based on chironomids are increasingly used and have been developed as a tool to track past changes in air temperature [3,10,16,26,27,28,29,30,31], such studies using modern geochronological and climate reconstruction models are still sparse in the Eastern Baltic region. Over the last few decades such studies were carried out in Poland [3,32,33], followed by reconstructions using data from Kurjanovas Lake in Latvia, Nakri Lake in Estonia [34,35], and Kamyshovoje Lake in Kaliningrad [30]. As the results obtained showed that reconstructions made with different models show slightly different results, the development of new models was launched, incorporating as much data as possible from the study areas [28,33,36]. In Lithuania, studies of chironomids and temperature reconstructions have only been conducted on the Lieporiai section in northern Lithuania [37]. Moreover, only a few quantitative climate reconstructions have been carried out in Lithuania so far, using pollen analysis data [35,38,39]. The need for such reconstructions is obvious, as data from all regions are needed to provide the most complete and detailed picture of climate change.

This paper aims to provide new information on palaeoecological conditions, with a particular focus on climate (quantitative inferences of mean July temperature), trophic state, acidification, and changes in water levels during the Early Holocene in southern Lithuania. Our goal is also to evaluate temperature variations that reveal regional and global climatic events. We hope that our studies will improve our understanding of the climate and environment of the Eastern Baltic during the Early Holocene.

## 2. Materials and Methods

### 2.1. Study Site

The Čepkeliai natural wetland complex is in the southern part of Lithuania. It is one of the largest bog complexes in Lithuania and Europe, and contains one of the few large, almost untouched raised bogs in the Baltic region [40]. The region is part of the boreo-nemoral vegetation zone dominated by *Picea abies*, *Pinus sylvestris*, *Betula pubescens*, and *Betula pendula* [41]. The Čepkeliai complex includes raised bogs (82% of the total area), lowland sedge bogs, black-alder swamps, forests, marsh islands, and 21 small wetland lakes. The largest part of the complex consists of a raised bog with an area of 5858 ha. It is a gently undulating plain with altitudes ranging from 128.5 to 134.4 metres above sea level, located on the marginal area of the Late Weichselian Glaciation [42].

**Figure 1:**
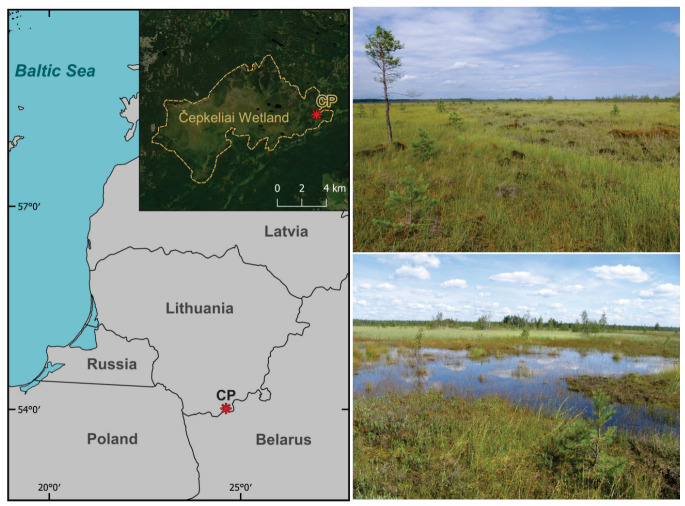
Location of the study site. Coring point (CP) marked with an asterisk. Photos of the Čepkeliai wetland: on the top is the coring point area, at the bottom is the coring point area after heavy rain (photo by G. Kibirkštis).

The investigated site (54°00″ N; 24°30″ E) is in the southeastern part of the Čepkeliai wetland, near the Lithuanian–Belarusian border, about 2.5 km from the Kriokšlys village (Figure 1). The site is a typical raised bog, with an elevation of about 131 m a. s. l. It is surrounded by a strip of birch thickets and a pine dominated forest [43]. The average temperature in July reaches up to +17.7 °C while in January it decreases to −5.4 °C. The average annual air temperature is +6.2 °C, and the annual mean precipitation in the region is around 673 mm [44].

### 2.2. Coring and Sampling

The sediment core was obtained in the southeastern part of Čepkeliai (54°00′48.54″ N, 24°37′1.02″ E), employing a “Russian” corer equipped with a 1 m-long inner chamber with a diameter of 5 cm. The lithology of sediment sequences was described visually in the field, wrapped into plastic tubes, and transported to the laboratory for subsampling. Eighty-one sediment sub-samples were taken every 4–6 cm for the Chironomidae survey.

The study presented here pertains to the sediment beds within the Early Holocene period, specifically the interval between 707 and 1150 cm in the sequence. Previous multiproxy studies including lithological (LOI, magnetic susceptibility), palaeobotanical (pollen, plant macrofossil, tree rings), isotopic (^14^C), and geochemical data are discussed in a paper by Stančikaitė [45].

### 2.3. Chronostratigraphy

**Figure 2:**
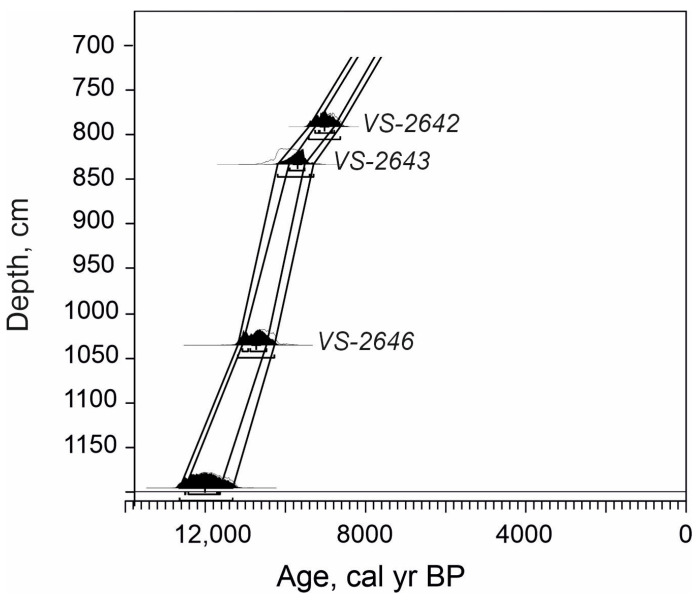
The age–depth model of Čepkeliai section (after Stančikaitė et al. [45]).

The age–depth model of the analysed sequence was adopted from Stančikaitė et al. [45] (Figure 2). The chronology of the investigated sequence is based on the four ^14^C dates obtained in the Laboratory of Nuclear Geophysics and Radioecology of the Nature Research Centre, Vilnius. The radiocarbon dates suggest that the investigated sediments under consideration were deposited during the Early Holocene, i.e., from ~11,200 cal yrs BP to ~7800 cal yrs BP.

### 2.4. Chironomidae Analysis

Treatment of the sediment samples for chironomid analyses followed Brooks [1]. A total of 81 wet samples were selected and analysed for their chironomid content (sample weight range from 1.0 to 8 g). Sediments were mechanically cleaned and deflocculated in a 10% potassium solution (KOH). Then, the samples were washed with distilled water, sieved through 200 and 90 µm sieves, and transferred to a Bogorov counting chamber [46]. Chironomidae larval head capsules were picked from the sorting dish under a stereomicroscope Nikon SMZ 1500, manufacturer: Nicon Europe B.V., Amstelveen, The Netherlands (a magnification of ×20–40), 1–2 head capsules were mounted on a standard microscope slide and covered with coverslips (0.01 mm thick and 6 mm in diameter). From each sample, a minimum of 50 head capsules were picked following methodical recommendations. Chironomidae taxa identification was performed under a biological microscope at ×200–×400 magnification with references to Wiederholm [47], Brooks et al. [1], and Larocque-Tobler [48].

### 2.5. Numerical Methods

**Figure 3:**
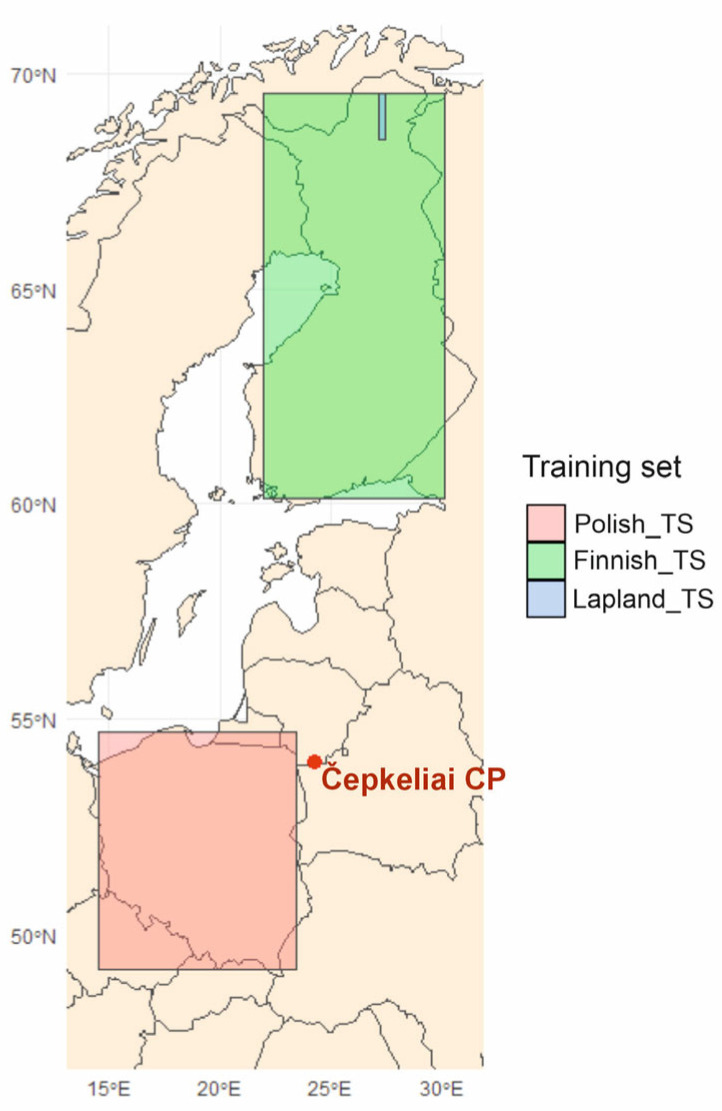
Location of the training sets used in this study (after [28,52]).

TILIA (version 1.0.1), TILIA GRAPH (version 2.1.1) [49], and Corel Draw 2024 software were used to construct the Chironomidae diagram. Cluster analysis grouped taxa into local Chironomidae zones using the (CONISS) [50]. Chironomidae assemblages were analysed using DCA (detrended correspondence analysis) to investigate the underlying trends within the data [51]. The palaeotemperature reconstructions were completed at Helsinki University by Dr. Tomi P. Luoto (the Faculty of Biological and Environmental Sciences, Department of Geosciences and Geography). Currently no chironomid-based climate calibration set is available from Lithuania, so two chironomid-based mean July air temperature inference models were used. The first is based on the expanded Fennoscandian training set (FTS) (weighted-averaging partial least squares, WAPLS) combining several data sets: Lapland and Finland [52] (Figure 3). The temperature gradient in this training set varies from 7.9 to 17.6 °C. The 2-component model includes 180 lakes and 129 taxa having an r^2^_jack_ of 0.86, a root mean squared error of prediction (RMSEP) of 0.85 °C, and a maximum bias of 0.75 °C. And the second Finnish–Polish training set (FPTS) uses the fossils data sets from Finland and Poland [28]. The combined calibration model includes 212 sites, 142 taxes, and a temperature gradient of 11.3–20.1 °C (Figure 3). The 2-component WAPLS model has a cross-validated coefficient of determination of 0.88 and a root mean squared prediction error of 0.88 °C. Sample-specific errors in the reconstruction were assessed using bootstrapping cross-validation with 999 iterations.

Pearson correlations were used to examine the relationships between the first two PC axes of each training set. The reconstruction for both training sets was examined using the modern analogue technique (MAT) to assess the representativeness of the used training set for the core data. The closest modern analogues (MinDC) were estimated using squared chi-square distances of the 10 closest analogues with 5 and 10 percentile thresholds for poor/very poor analogues. In addition, the response of chironomids to the reconstructed variable was estimated by calculation, the Pearson product–moment correlation coefficient between chironomid principal component axis 1 scores, and the reconstructed temperature values in principal component analysis (PCA). The PCA was run using square-root transformed species data. To perform numerical analysis and data plots, the R software (version 4.1.1.) was used with the following packages: for statistical analysis: “stat” [53], visualisation: ”corrplot” [54], ggplot2 [55], ”rnaturalearth [56], ”rnaturalearthdata” [57].

## 3. Results

### 3.1. Chironomidae Assemblages

**Figure 4:**
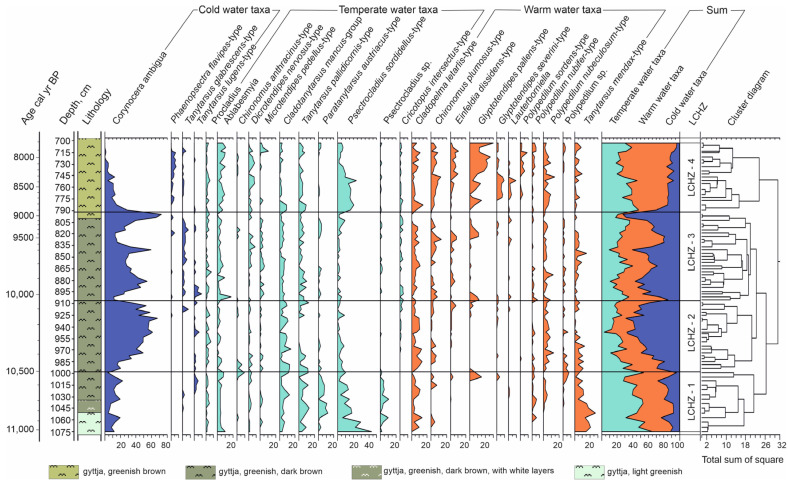
Percentage diagram of Chironomidae assemblages for most abundant taxa.

Chironomidae were analysed in the sediment interval 1075–703 cm. The most abundant taxa were *Corinocera ambigua*, *Cladopelma lateralis*-type, *Tanytarsus mendax*-type, *Polypedilum nubeculosum*-group, and *Cladotanytarsus mancus*-group. Rare taxa with abundance ˂3% were *Microchironomus*, *Micropsectra pallidula*-type, *Psectrocladius barbatipes*-type, *Endochironomus impar*-type, *Cricotopus* sp., *Cladopelma laccophila*-type, and *Tanytarsus nemorosus*-type. Four statistically significant local Chironomidae zones (LCHZ) were distinguished according to the variation in chironomids morphotype composition throughout the section (Figure 4).

The first LCHZ (~11,000–10,500 cal yr BP (1075−1000 cm)) is dominated by temperate-water taxa, accounting for up to 30–54% of the assemblage, followed closely by warm-water taxa (~25–41%). Cold-water taxa are present in lower proportions. Of the taxa found in temperate waters, the *Psectrocladius sordidellus*-type is the most abundant, reaching numbers as high as 40%. Other temperate-water taxa, such as *Cladotanytarsus mancus*-group, *Paratanytarsus austriacus*-type, and *Tanytarsus pallidicornis*-type, range from 2% to 12%. The warm-water indicator *Tanytarsus mendax*-type reaches abundances of up to 27%. Among the cold-water taxa, *Corynocera ambigua* predominates, reaching up to 20%, while *Tanytarsus glabrescens*-type and the cold-water indicator *Tanytarsus lugens*-type are found in very small numbers in the upper part of this zone.

The second LCHZ (10,500–10,050 cal yr BP (1000–905 cm)) is characterised by a significant increase in cold-water taxa, which reach their highest values throughout the section (~60%). Warm-water taxa decrease to ~32%, while temperate-water taxa decline to approximately 10–23%. The cold-water taxon *Corynocera ambigua* is the most abundant, reaching up to 65% of the total. *Tanytarsus lugens*-type fluctuates between 2% and 4%. Among the warm-water taxa *Cladopelma lateralis*-type predominates, reaching up to 15% while *Polypedilum nubifer*-type and *Tanytarsus mendax*-type comprises about 2–10%. Temperate-water taxa show some compositional changes: the *Cladotanytarsus mancus*-group is dominant, reaching its maximum at around 15%, Ablabesmyia fluctuates around 3%, and *Tanytarsus pallidicornis*-type fluctuates at around ~5%. There is a considerable decrease in Psectrocladius *sordidellus*-type (~2–4%) and *Paratanytarsus austriacus*-type.

The cold-water taxa are still the most common in the third LCHZ (10,050–9000 cal yr BP (905–792 cm)), making up around 40% and increasing up to 80% at the end of the zone. However, their share of the total slightly decreases from the second zone. Cold-water taxon *Corynocera ambigua* still dominates (20–80%), and *Tanytarsus glabrescens*-type fluctuates from 2% to 6%. *Tanytarsus lugens*-type reaches up to 7% at the beginning of the zone but then almost disappears, occurring only sporadically at around 1%. There is a recorded increase in warm-water taxa such as *Chironomus plumosus*-type and *Einfeldia dissidens*-type. Their abundances fluctuate between 3% and 10%. The proportion of temperate-water taxa remains nearly unchanged and is very similar to that observed in the second LCH zone. However, there is slight decrease in *Psectrocladius sordidellus*-type (~2%), while Ablabesmyia slightly increases (2%–10%).

The fourth LCHZ (9000–7800 cal yr BP (792–703 cm)) marks a transition to warm-water taxa dominance up to 56%, while temperate-water taxa remain substantial, and cold-water taxa decline on average to about 12%. The dominant warm-water taxa are *Glyptotendipes pallens*-type (~5–30%), *Polypedilum nubeculosum*-type (~2–15%), *Chironomus plumosus*-type (~2–12%), and *Cladopelma lateralis*-type (~5–20%). Temperate-water taxa remain abundant, with *Psectrocladius sordidellus*-type being the most numerous, reaching up to 20%, followed by *Cladotanytarsus mancus*-type and Ablabesmyia at around 10% each. Among the cold-water taxa, *Tanytarsus lugens*-type disappears completely, while *Corynocera ambigua* decreases from 40% to 2%. Notably, the abundance of *Phaenopsectra flavipes*-type reaches its maximum in this zone at 5%.

### 3.2. Statistical Analysis

**Figure 5:**
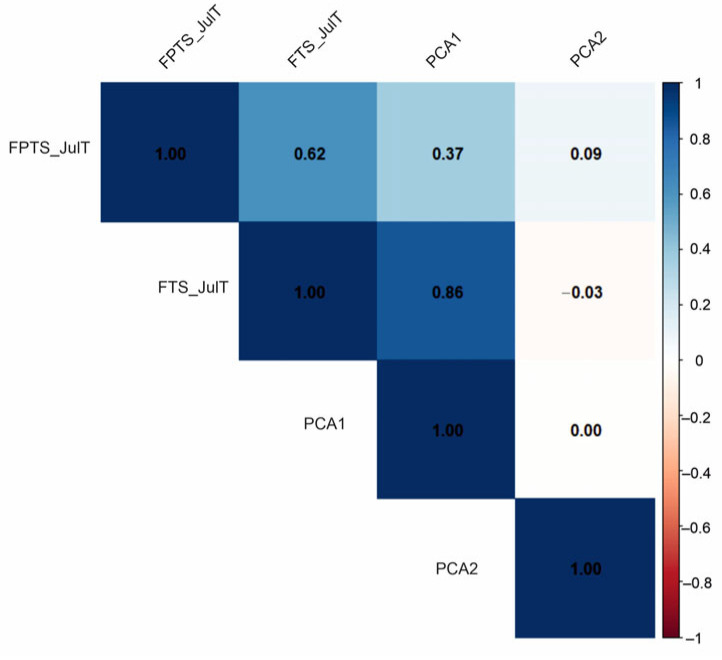
Pearson correlation coefficient matrix.

The initial detrended correspondence analysis (DCA) showed that the obtained axis length was 2.5 SD. Therefore, the chironomid data were analysed using a linear ordination method (PCA). As results, the matrix presents Pearson’s rank correlation between the principal components PC1 and PC2 derived from chironomid and temperature reconstruction indicators (July temperatures and root-mean-square error of prediction (RMSEP) calculated using the FPTS and FTS). Strong positive correlations (blue) indicate variables that change in the same direction, while strong negative correlations (red) indicate opposing trends. The first principal component (PC1) axis of chironomid assemblages is strongly correlated (|r| > 0.6) with the FTS July temperature; meanwhile, the correlation with the FPTS July temperature is very weak (|r| < 0.6). In contrast, the second principal component (PC2) axis shows a weak or even negative correlation (Figure 5).

The reconstructed temperatures showed different levels of agreement with the chironomid PCA1 axis depending on the sample group. For the FPTS samples, the correlation was relatively weak (R = 0.37, r^2^ = 0.14, *p* < 0.001), indicating that only 14% of the variation in PCA1 is explained by the reconstructed temperatures. In contrast, the FTS samples exhibited a much stronger correlation (R = 0.86, r^2^ = 0.73, *p* < 0.001), with 73% of the PCA1 variation explained. This demonstrates that while the general trends are captured in both groups, the accuracy of individual reconstructed values is substantially higher in FTS due to better-quality modern analogues.

**Figure 6:**
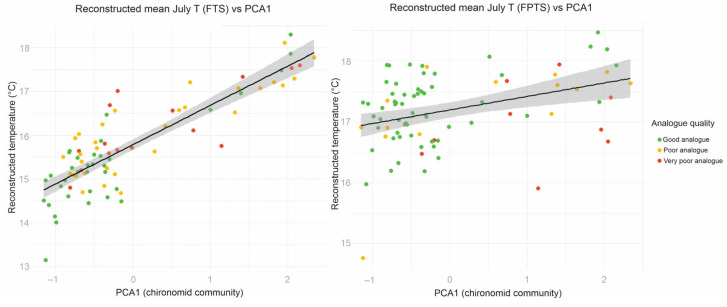
Relationship between reconstructed July temperature (left: FTS; right: FPTS) and PCA1 (chironomid community) with modern analogue assignment (MinDC). The grey shading represents the 95% confidence interval around the regression line, illustrating the uncertainty of the modeled relationship.

The training sets were compared based on bootstrapped sample-specific errors (eSEP) and closest modern analogues (MinDC) data. In the FPTS, the eSEP in the reconstruction varied between 0.91 and 0.98 °C, suggesting that none of the samples had a high risk of being erroneous. Although all of the core taxa were present in the training set, 9 of the total 81 samples had a very poor modern analogue based on the MAT, whereas 14 additional samples had poor modern analogues. The 58 good analogues were at sample depths 703–715 cm, 729 cm, 751 cm, 769 cm, 783–789 cm, 799 cm, 807–815 cm, 823–827 cm, 837–849 cm, 857–861 cm, 873–895 cm, 909–913 cm, 921–965 cm, 973–999 cm, and 1009–1075 cm. However, despite the generally poor modern analogues, the reconstructed values correlated strongly and statistically significantly with the chironomid primary PC axis values (R = 0.37, r^2^ = 0.14, *p <* 0.001) (Figure 6).

In the FTS, the eSEP in the reconstruction varied between 0.86 and 0.92 °C, suggesting that none of the samples had a high risk of being erroneous. Although all of the core taxa were present in the training set, 15 of the total 81 samples had a very poor modern analogue based on the MAT, whereas 31 additional samples had poor modern analogues. The 35 good analogues were at sample depths 703 cm, 725–729 cm, 747–751 cm, 789–795 cm, 853–861 cm, 889 cm, 909–913 cm, 921 cm, 929 cm, 949–953 cm, 965–985 cm, 993 cm, 1009 cm, 1027–1039 cm, 1051 cm, and 1063–1075 cm. However, despite the generally poor modern analogues, the reconstructed values correlated strongly and statistically significantly with the chironomid primary PC axis values (R = 0.86, r^2^ = 0.73, *p <* 0.001). This confirms that the reconstructed trends are reliable, but the poor modern analogues might decrease the accuracy of the specific reconstructed values (Figure 6).

### 3.3. Chironomid-Inferred Palaeotemperatures (T^Jul^)

**Figure 7:**
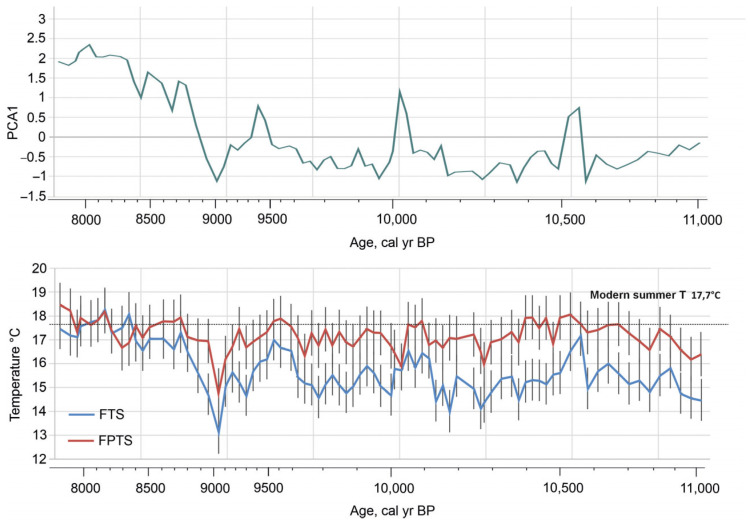
Chironomid-inferred palaeotemperature (T^Jul^) reconstructions from Čepkeliai bog using Fennoscandian and Finnish–Polish calibration data sets [26,28].

Overall, the reconstructed temperatures vary within a similar range for both models: between 13.2 °C and 18.5 °C based on FTS and between 14.6 °C and 18.5 °C based on FPTS (Figure 7). Each reconstruction shows a series of short-term temperature variations. Some of these overlap, but there are also differences. In the lower part of the section, at about 11,000 cal yrs BP, average July temperatures may have been around 14.5 °C (FTS) and 16.2 °C (FPTS). Shortly afterwards, palaeotemperatures began to rise, increasing by 2–3 °C. The maximum temperature recorded during the Preboreal period (11,500–9900 cal yrs BP) was 17.5 °C, as determined by the FTS, and 18 °C, as indicated by the FPTS at approximately 10,575–10,540 cal yrs BP. Both reconstructions then show a temperature drop to 14.2 °C degrees for the FTS and to 16 degrees for the FPTS at about 10,250 cal yrs BP. This climate instability can be attributed to the “10.2 oscillation”. This drop in temperature was followed by a short rise and then a drop again to 16 °C for the FTS at 10,185 cal yrs BP and to 15.8 °C for the FPTS at 10,085 cal yrs BP. Later, the temperature began to rise, reaching 17 °C (FTS) and 17.9 °C (FPTS) at around 9500–9600 cal yrs BP. Shortly thereafter, this increase is followed by a gradual decrease in temperature until it drops to 13 °C (FTS) and 14.6 °C (FPTS) at around 9000 cal yrs BP. After this cold event, according to both temperature reconstructions, the July temperature increases again and fluctuates between 16.5 and 18.5 °C in the upper part of the record.

## 4. Discussion

### 4.1. Climatic Variation and Related Environmental Changes

The investigation’s findings enable a detailed description of the local environmental and ecological conditions, as well as the development stages of the Čepkeliai palaeobasin.

**Figure 8:**
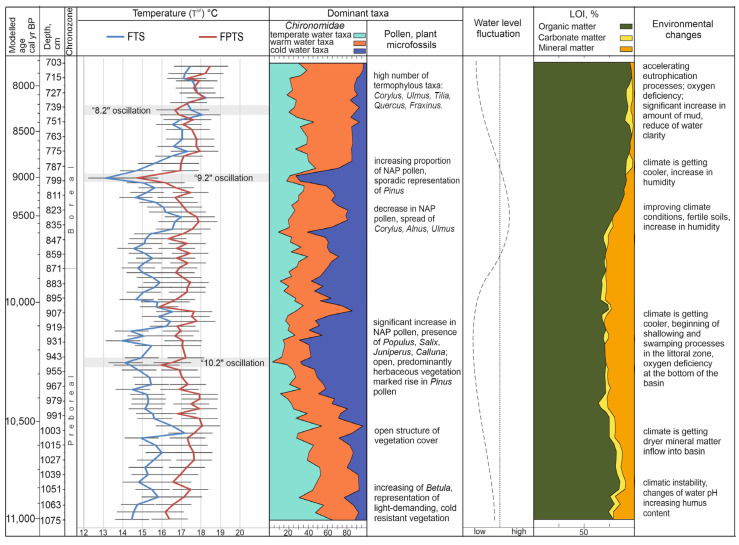
Summary chart of reconstructed palaeoenvironmental changes from Čepkeliai bog. Pollen, plant macrofossil, and LOI data were cited from Stančikaitė et al. [45].

At the beginning of the Early Holocene, around 11,000 cal yrs BP, the Čepkeliai palaeobasin was dominated by Chironomidae taxa characteristic of mesotrophic to eutrophic water basins, which are found in shallow or medium-depth habitats (Figure 8). The low water level of the palaeobasin is also evident from the presence of macrofossils of *Menyanthes trifoliata* and *Carex* sp., as well as the growth of macrophytes in the littoral zone [45]. Taxa of the *Cladotanytarsus mancus*-group, along with the representatives of the *Polypedilum* genus, are thermophilic indicators of warm waters [1]. The abundance of the *Psectrocladius sordidellus*-type may be related to an increase in the lake’s humus content and a change in its pH. [58]. The *Ablabesmyia* and *Psectrocladius* morphotypes are found in organic saturated sediments [59,60]. The presence of this Chironomidae composition indicates relatively warm climatic conditions and nutrient-rich sediments in the palaeobasin. However, the *Cladopelma laterallis* and *Paratanytarsus austriacus* types are common in water basins with few macrophytes and tolerate sand and gravel sediments [1]. The presence of such a diverse Chironomidae fauna indicates unstable environmental conditions, as climatic fluctuations were common during the Preboreal period [61]. According to the FTS, the mean July temperature may have fluctuated around 14.5–15 °C during this period, whereas it was 1.5 °C higher under the FPTS.

From ~10,600 to 10,100 cal yrs BP, the cold-adapted taxon *Corynocera ambigua* became increasingly prevalent. The number of taxa characteristic of warm waters is decreasing. Morphotypes characteristic of the littoral zone are flourishing. This change in chironomid composition may indicate arid conditions and a low water level in the palaeobasin, as representatives of the genus *Microtendipes*, which indicate low water levels, have been observed [62]. Fluctuations in the water level during this period may have been one of the most important factors contributing to the change in morphotype composition of the Chironomidae in the Čepkeliai palaeobasin. The presence of numerous taxa inhabiting sandy sediments, such as the *Cladotanytarsus mancus* group and the *Cladopelma lateralis* type, suggests the occurrence of mineral material that may have been transported into the basin by erosion processes or inflowing water [63]. The increase in the amount of mineral material is confirmed by the sediment composition studies (Figure 8). According to the FTS, the mean July temperature may have fluctuated around 14.2–17.2 °C and 16.8–18 °C under the FPTS.

At about 10,300–10,000 cal yrs BP some increase in cold-adapted *Tanytarsus lugens*-type is observed. The abundance of *Chironomus plumosus*-type taxa indicates a lack of oxygen at the bottom of the lake, as this genus tolerates low-oxygen, mesotrophic–eutrophic environmental conditions [64,65]. However, the oxygen concentration was sufficient for the predatory representatives of the *Procladius* and *Ablabesmyia* morphotypes to survive, as oxygen is necessary for their development [14]. These ecological conditions are confirmed by an increase in herb pollen, indicating an open vegetation cover during the period of 10,400–10,200 cal yrs BP [45]. Pine is also an important component of the vegetation, suggesting the presence of sandy soils. The flourishing of the aquatic macrophytes such as *Najas alba* and *N. marina* as well as recorded *Potamogeton* species suggests shallow water conditions. These changes in the vegetation correlate perfectly with the results of Chironomidae studies proving the onset of the shallowing and swamping processes in the Čepkeliai palaeobasin. According to the FTS, the chironomid-inferred mean July temperature during this period drop to ~14 °C. Under the FPTS, however, it was 1.7 °C higher. This change in climatic conditions is possibly related to the “10.2” climate oscillation, registered in many lacustrine, tree-ring, ice-core, and marine records in the North Atlantic and Europe [66]. Further development of the Chironomidae composition shows the increase in taxon diversity at ~10,000–9300 cal yrs BP. The warm-adapted taxa are dominated by the mesotrophic–eutrophic morphotypes. The prevalence of the cool-water genus *Procladius* suggests a rise in water level, which correlates well with the increase in pollen of swamp water plants and the abundance of alders, which require wet habitats [45]. The spread of broad-leaved trees such as linden and oak also indicates changes in the climatic conditions. During this period, the mean July temperature according to Chironomidae data could have reached more than 17 °C under the FTS and about 18 °C under the FPTS (Figure 8).

However, this temperature rise has not been continuous. Subsequent changes in the chironomid record indicate some climatic changes at ~9300 cal yrs BP to 9000 cal yrs BP. Significant changes in the chironomid fauna are observed, such as a decrease in the fossil record of the warm-adapted tribe Chironomini and a significant increase in the number of cold-adapted *Corynocera ambigua*. A marked drop in temperature of 3–4 °C is observed when the temperature falls from 17 to 13.1 °C (according to FTS) and 17.8 up to 14.7 °C (according to FPTS). This cooling off period may be related to the widespread 9.3 event which has climatic anomaly patterns very similar to the 8.2 ka event [67].

Shortly thereafter, between 9000 and 7800 cal yrs BP, the largest increase in the number of warm-adapted Chironomini tribe taxa in the history of palaeobasin development was recorded, as well as an increase in the number of the littoral and sublittoral morphotypes of the genera *Polypedilum*, *Glyptotendipes*, and *Cladopelma*. This proves the acceleration of eutrophication processes and the oxygen deficiency in the bottom zone of the lake [68]. An increase in silt and a decrease in water clarity changes the diversity of littoral Chironomidae morphotypes. The growing number of cool-water *Ablabesmyia* taxa confirms an increase in nutrients, as these taxa feed on zoobenthos. Representatives of the Chironomini tribe indicate eutrophic conditions and poor water quality [69]. Overall, the amount of minerogenic additions decreased, and organic material accounted for more than 90% of the total (Figure 8). The deposition rate dropped down twice [45]. According to both chironomid data sets, mean July temperatures could have reached as high as about 18.5 °C. Changes in vegetation, such as the spread of deciduous trees with higher thermal requirements, such as *Ulmus*, *Fraxinus,* and *Quercus*, in the surrounding area confirm an improvement in climatic conditions [45] (Figure 8).

### 4.2. Temperature (T^Jul^) Variations During the Early Holocene in a Regional Context

Lacustrine records from northern and central Europe indicate the hydrological and biological impacts of climate change at the beginning of the Holocene period. Quantitative reconstructions of summer temperatures based on chironomid assemblages reveal an increase of up to 6 °C across the Younger Dryas/Holocene boundary, with the greatest changes experienced in the mid-latitudes (50–60°N) and along the Atlantic margins of Western Europe [34]. Although the Holocene showed less climate variability than the Lateglacial, it was not stable either. The Early Holocene is marked by several centennial-scale cooling anomalies: the Preboreal Oscillation (PBO) [70,71]; the 10.2 ka event [66]; the 9.3 ka (or, 9.2 ka) event [67]; and the 8.2 ka event [72], which defines the Early/Middle Holocene boundary. However, not all these climatic variations are reflected equally clearly in temperature reconstructions, with some being more accurately represented than others.

Until now, only one chironomid-based palaeotemperature reconstruction had been carried out in Lithuania: at the Lieporiai site in the north of the country [37]. Pollen-based palaeotemperature reconstructions have been performed at four sites: Lieporiai (N Lithuania), Čepkeliai (S Lithuania), Dūkštelis (ES Lithuania) [39], and Petrašiūnai (N Lithuania) [35,39]. Some detailed chironomid-based palaeotemperature reconstructions are available from the Baltic region [3,28,30,35].

The estimated mean July air temperature for the Early Holocene at the Čepkeliai site ranged from 13.2 °C to 18.5 °C (FTS) and from 14.6 °C to 18.5 °C (FPTS). Pollen-based mean summer temperature reconstructions from the Early Holocene period at the Čepkeliai palaeolake shows temperature ranging from 11 to 16 °C [39]. The chironomid-based reconstructions from the Lieporiai site (FTS) ranged in the interval 14–15.5 °C [37], meanwhile pollen-based reconstruction at this site ranged from 13 to 16 °C. The Petrašiūnai reconstruction ranged from 11.5 to 18 °C. Respectively, the reconstructions from the north Baltic areas of Latvia, Kurjanovas Lake show ~12–17 °C and Estonia, Nakri Lake show ~11.2–17 °C [35]. Similar temperatures were recorded in the south Baltic areas of Kamyshovoye Lake, Russia, ranging ~12.5–16 °C [30] and Poland, Žabieniec bog ~14–15 °C.

Our study recorded three cold events on top of the general warming trend. The first drop in temperature of up to 1.5 °C was recorded at around 10,300–10,200 cal yrs BP. This change in climatic conditions can be attributed to the “10.2” climate oscillation [66]. In the Lieporiai chironomid-based temperature reconstruction, this event is not clearly distinguished due to the reason of slow sedimentation rate [37]. According to Kamyshovoye data, the temperature dropped from 14.2 °C to 13.4 °C [30]. In the Žabieniec bog reconstructions, however, this is not visible [3]. Temperature reconstructions based on chironomids at Hawes Water Lake (northwest England) show a fall of 1.2 °C around 10.4 cal BP [5].

A notable drop in temperature is observed at around 9000–9100 cal yrs BP, when the temperature decreased from 17 to 13.1 °C (according to FTS) and from 17.8 to 14.7 °C (according to FPTS). This climate cooling may be related to the widespread “9.3” climate oscillation [73]. Meanwhile, this event is not fixed in the Lieporiai temperature reconstruction, and this may be caused by the lowering of water and very slow sedimentation [37]. However, this is also not reflected in the temperature reconstruction based on chironomids in the Kamyshovoye section. This may have been influenced by the low water level in the palaeobasin, as confirmed by diatom and geochemical studies [30]. Numerical analysis of the diatoms in Kašučiai (Lithuania) and Kamyshovoye (Kaliningrad region) sections revealed that the “9.3” ka cooling event caused a decrease in the diversity of freshwater diatoms [74]. Chironomid based temperature reconstructions, fixed a cooling of 2.2 °C for the 9.3 ka even at Hawes Water Lake in northwest England [5,7].

The “8.2” ka event is less prominent in our reconstructions compared with the “9.3” ka event. It is better reflected in Finnish–Polish TS reconstruction where the temperature dropped by 1.5 °C. Meanwhile according to Fennoscandian TS reconstruction, the temperature fell by 0.5 °C. At Žabieniec, inferences based on the Swiss and Russian data sets indicate a cold event between 8.7 and 8.0 ka BP. Smooth negative temperature variation is fixed in the Kamyshovoye temperature reconstruction shortly after the 8.2 ka event [30].

## 5. Conclusions

Chironomid studies at the Čepkeliai bog palaeobasin have offered an opportunity to reconstruct the environmental history of the site during the Early Holocene, with a particular focus on the quantitative reconstruction of palaeotemperature. Climatic variations were reconstructed based on two calibration data sets (Fennoscandian and Finnish–Polish). This new information provides a valuable addition to the scarce existing records from the Eastern Baltic region.

The reconstructed mean July temperatures ranged from 13.2 °C to 18.5 °C based on Fennoscandian TS, and from 14.6 °C to 18.5 °C based on Finnish–Polish TS. Although a rise in temperature of about 4–5 °C was reconstructed towards the top of the section, both reconstructions capture a series of climatic events that occurred during the Early Holocene, including the “10.2”, “9.2”, and “8.2” short-term oscillations. Comparison of estimated temperatures from Čepkeliai with those from adjacent regions shows that the amplitude of temperature change is consistent.

Throughout its history, the palaeobasin has undergone a series of environmental changes that have led to shifts in chironomid communities. While temperature is a decisive factor in chironomid development, this study shows that local environmental conditions such as water level, eutrophication, and pH changes are also significant factors. Together with global processes, these factors may be responsible for differences in how natural systems respond to global trends.

Our research shows the potential of chironomid analysis in identifying rapid, short-lived climatic shifts during the Holocene but also highlights the need for further high-resolution studies using the chironomid data set from Lithuania.

## Data Availability

The data presented in this study are available on request from the corresponding author. The data are not publicly available because they form part of an ongoing research project and will be used for subsequent analyses and future publications.
